# HMGN5 Silencing Suppresses Cell Biological Progression via AKT/MAPK Pathway in Human Glioblastoma Cells

**DOI:** 10.1155/2020/8610271

**Published:** 2020-05-21

**Authors:** Quanfeng Ma, Xiuyu Wang, Hong Wang, Wen Song, Qiong Wang, Jinhuan Wang

**Affiliations:** ^1^Tianjin Key Laboratory of Cerebral Vascular and Neurodegenerative Diseases, Tianjin Neurosurgical Institute, Department of Neurosurgery, Tianjin Huanhu Hospital, Tianjin 300350, China; ^2^The Graduate School, Tianjin Medical University, Tianjin 300070, China; ^3^Department of Neurosurgery, Tianjin First Center Hospital, Tianjin 300192, China

## Abstract

HMGN5 regulates biological function and molecular transcription via combining with a nucleosome. There has been growing evidence that aberrant expression of HMGN5 is associated with malignant neoplasm development and progression. In the present study, we found that the expression of HMGN5 is significantly higher in high-grade glioblastoma tissues than in low-grade samples. To clarify the function of HMGN5 in glioblastoma, we knocked down HMGN5 in U87 and U251 glioblastoma cells via siRNA. The results demonstrated that HMGN5 was involved in the regulation of proliferation and apoptosis, migration, and invasion of glioblastoma cells. These outcomes also indicated that silencing HMGN5 possibly suppressed the expression of p-AKT and p-ERK1/2. Taken together, our research reveals that HMGN5 might be an efficient target for glioblastoma-targeted therapy.

## 1. Introduction

Glioblastoma is the most common intracranial tumor which developed from the supporting nerve cells, neuroglia [1]. According to World Health Organization (WHO) criteria, glioblastoma was histologically classified into grades I to IV [2]. The most malignant subtype of these is glioblastoma (GBM) (grade IV), whose survival is shorter than 2 years.The current treatment for glioblastoma is surgical resection combined with radiotherapy and/or temozolomide chemotherapy. However, due to the invasiveness and recurrence of this tumor, the therapeutic efficacy and prognosis of patients with glioblastoma are always unsatisfactory [3]. Therefore, an increasing number of researches have focused on the molecular biology of glioblastoma to search for novel therapeutic strategies.

King and Francomano first reported the HMGN5, and they found that it was mainly expressed in the nucleus of cells [4, 5]. As a member of the high-mobility group protein (HMGN), HMGN5 can change the structure of chromatin and regulate the biological function and molecular transcription by combining with a nucleosome [6]. The oncogenic potential of this protein was found gradually in many types of human tumors. A higher expression level of HMGN5 was found in cancerous tissues of prostate cancer, bladder cancer, renal cancer, and breast cancer compared to their respective nontumor tissues. And silencing HMGN5 could suppress cell proliferation and invasion as well as induce cell apoptosis of tumor cells mentioned above [7]. Studies showed that knockdown of HMGN5 could sensitize several tumor cells to radiotherapy or some chemotherapeutic drugs. For instance, silencing HMGN5 increased the sensitivity of prostate cancer cells to ionizing radiation [8]. The cisplatin resistance of bladder cancer could be regulated by HMGN5 through PI3K/AKT signaling [9]. Except for PI3K/AKT, it was reported that HMGN5 promoted proliferation and invasion of pancreatic ductal adenocarcinoma through the Wnt/*β*-catenin signaling pathway [10]. In glioblastoma, although previous research showed that the overexpression of HMGN5 promoted proliferation and survival of glioblastoma cells [11], the mechanisms underlying the oncogenic role of HMGN5 remained unclear. Moreover, whether HMGN5 is related to the aggressiveness and chemoresistance of glioblastoma needs further elucidation.

In this study, we investigated the expression level of HMGN5 in glioblastoma tissues and cell lines. The effects of HMGN5 on proliferation, apoptosis, migration, and invasion of glioblastoma cells were observed by RNA interference to silence this gene in vitro. Our data proposed HMGN5 as a critical molecule which is involved in the regulation of malignant behavior as well as in the PI3K/AKT and MAPK pathway in GBM cells, suggesting that this protein might be a possible target for glioblastoma-targeted therapy.

## 2. Materials and Methods

### 2.1. Clinical Samples

There were 5 glioblastoma tissues and 5 nontumoral brain tissues obtained from the Department of Neurosurgery, Tianjin Huanhu Hospital. The 5 nontumoral brain tissues were obtained from patients with severe traumatic brain injury. The protocol for using patient samples was approved by the ethics committee of Tianjin Huanhu Hospital, and informed consent was obtained from all the patients and control individuals according to the Declaration of Helsinki.

### 2.2. Cell Culture and Transfection

Six glioblastoma cell lines (SNB19, A172, LN229, LN308, U251, and U87) were bought from the American Type Culture Collection (Genetimes ExCell Technology, Inc., Shanghai, China). All the cells were cultured and maintained in Dulbecco's modified Eagle's medium (DMEM; Gibco, USA) supplemented with 10% fetal bovine serum (FBS, Gibco, USA) in a humid atmosphere at 37°C with 5% CO_2_.

Cells were transfected with small interfering RNA (siRNA) at 50% confluency using Lipofectamine 2000 (Invitrogen, California, USA) according to the manufacturer's recommendations. The HMGN5-siRNA and scramble sequences were synthesized by Gima Biol Engineering Inc. (Shanghai, China):

(i) HMGN5-siRNA
  - 5′-CACAGCCTTTCTTTAGCATTT-3′ (sense)
  - 5′-GTGTCGGAAAGAAATCGTATT-3′ (antisense)
(ii) Scramble sequence
  - 5′-UUCUCCGAACGUGUCACGUTT-3′ (sense)
  - 5′-GTGTCGGAAAGAAATCGTATT-3′ (antisense)

### 2.3. Extraction of mRNA and RT-qPCR

Total RNA was extracted from tissues and cells with the TRIzol Reagent (Invitrogen, California, USA). And then, the total RNA was reverse transcribed by the PrimeScript™ RT reagent kit (TaKaRa, RR037A, Japan) to obtain cDNA. Real-Time Quantitative Polymerase Chain Reaction (RT-qPCR) was performed using the Power SYBR Green PCR Master Mix (Applied Biosystems, Carlsbad, USA) on a LightCycler 480 II PCR machine (Roche, Basel, Switzerland) to quantify the expression of HMGN5 mRNA. *β*-Actin was used as an endogenous control. The primer pairs were as follows:

(i) HMGN5:
  - 5′-GGTTGTCTGCTATGCTTGTG-3′ (forward)
  - 5′-ACTGCTTCTTGCTTGGTTTC-3′ (reverse)
(ii) *β*-Actin:
  - 5′-CACCATGAAGATCAAGATCATTGC-3′ (forward)
  - 5′-GGCCGGACTCATCGTACTCCTGC-3′ (reverse)

### 2.4. Extraction of Protein and Western Blot

Tissue protein was extracted with a protein extracting reagent (BioTeke Corporation, Beijing, China) according to the manufacturer's directions, while cell protein was extracted through RIPA buffer supplemented with 1% protease inhibitors (Roche, Basel, Switzerland). After centrifugation, the protein content was measured by a BCA Protein Assay Kit (Solarbio, Beijing, China). According to the standard procedures of western blot, total proteins were separated by SDS-PAGE and transferred to PVDF membranes (Millipore, Bedford, MA, USA), and then blocked with 5% skim milk in TBST. After being incubated with antibodies, the membranes were visualized with the ECL procedure (Millipore, USA) to get protein bands, which were analyzed by ImageJ software. The primary antibodies included anti-HMGN5, anti-Bcl-2, anti-Bax, anti-Cyclin D1, anti-p21, anti-MMP-2, anti-MMP-9, anti-AKT, anti-p-AKT, anti-ERK1/2, anti-p-ERK1/2 (Santa Cruz Biotechnology, Inc., USA), and anti-*β*-actin (WanleiBio, Shenyang, China). The secondary antibodies included HRP-conjugated goat anti-rabbit IgG and goat anti-mouse IgG (OriGene Technologies, USA).

### 2.5. CCK8 Assays

CCK8 assays were used to detect the viability and proliferation of U87 and U251 cells with or without HMGN5 siRNA transfection. Briefly, at 24 h, 48 h, and 72 h posttransfection, the medium in the 96-well plates was changed to the CCK8 reagent, then the absorbance was measured with a microplate reader at 490 nm. The same volume of PBS buffer was added to wells without cells as a blank. The procedure was repeated three times for each group.

### 2.6. Flow Cytometry Analysis

For cell cycle assays, cells were collected at 48 h after being transfected with siRNAs or scrambled and fixed in 70% ice-cold ethanol overnight. After washing with cold PBS, the fixed cells were stained with propidium iodide (PI, Calbiochem) for 30 minutes in the dark. The washed cells were collected at 48 h after being transfected with siRNAs or scrambled sequences, the rate of apoptosis was detected by FCM with Annexin V-FITC and PI apoptosis detection kit (WanleiBio, Shenyang, China). Cells used to measure apoptosis rate and mitochondrial membrane potential (Δ*ψm*) were also collected at 48 h posttransfection. And the mitochondrial membrane potential (Δ*ψm*) of cells was detected by the JC-1 Kit (Beyotime, China). After washing with PBS twice, the harvested cells were incubated in the JC-1 dye for 30 min at 37°C with 5% CO_2_ in the dark. All the stained cells were measured on the FACSCanto II flow cytometer (BD Biosciences, USA) to get the data of DNA content and cell apoptosis rate. The FlowJo software was also used to analyzed the data mentioned above. The experiments were performed in triplicate.

### 2.7. Transwell Assays

Transwell assays were conducted to compare the motility and invasiveness of cells in the 3 groups. 5 × 10^3^ cells in 200 *μ*L serum-free medium were seeded onto Transwell chambers (Corning, Cambridge, USA) with or without a Matrigel-coated membrane. Matrigel was bought from BD Biosciences (Franklin Lakes, NJ, USA). The lower compartments of the chambers were filled with 500 *μ*L DMEM containing 10% FBS as the chemoattractant. After 24 hours of incubation at 37°C, the upper chambers were gently wiped by a cotton swab and the lower chambers were fixed with ethanol and stained with 1% crystal violet (Sigma-Aldrich, St. Louis, MO, USA) to count the cells that migrated or invaded to the lower surface. Eventually, the staining cells were counted in five different fields of view under an inverted fluorescence microscope (Nikon TE2000, Japan). Every experiment was performed in triplicate and repeated at least thrice.

### 2.8. Wound-Healing Assays

Glioblastoma cells from different groups were seeded on 6-well plates. The confluent cell layers were gently scratched by a 200 *μ*L sterile pipette tip to make linear wounds. Then, the cell layers were washed by fresh media to remove cell debris. Cells were stained by Hoechst 33258 dye and photographed with an inverted fluorescence microscope (Nikon TE2000) at 0 h, 12 h, 24 h, and 48 h after having been scratched. The proportion of wound healing was calculated by the following formula: 100% − (width of each time/width at 0 h) × 100%. Experiments were carried out at least in triplicate, and three random fields of each well were recorded.

### 2.9. Xenograft Experiments

All animal protocols were performed under the approval of the Animal Care and Use Committee of Tianjin Huanhu Hospital. Male athymic BABL/c nude mice (4 weeks old) were purchased from the PLA Military Academy of Medical Sciences Laboratory Animal Center (Beijing, China). The U87 subcutaneous tumor xenograft model was established before the experiment. When the tumor volume reached an average of about 100 mm^3^, the male nude mice were randomly divided into two groups, which were injected with HMGN5 siRNA or scramble sequences into the xenograft model through intratumoral injection of the xenograft tumor every 3 days while tumor volume was measured (volume = (length × width 2)/2). The xenograft growth curves were based on the mean volume of each group, and inhibition was calculated. Twenty-one days after the inoculation, the mice were killed and the tumors were stored in liquid nitrogen and formalin according to standard procedures.

### 2.10. Statistical Analysis

All data were presented as mean ± standard deviation (SD) values. GraphPad Prism 6.0 (CA, USA) and SPSS 22.0 were used to perform statistical analyses. One-way ANOVA or Student's *t*-test was used for comparisons between groups. *P* < 0.05 was determined as statistically significant.

## 3. Results

### 3.1. HMGN5 Is Upregulated in Glioblastoma Tissues and Cells

**Figure 1:**
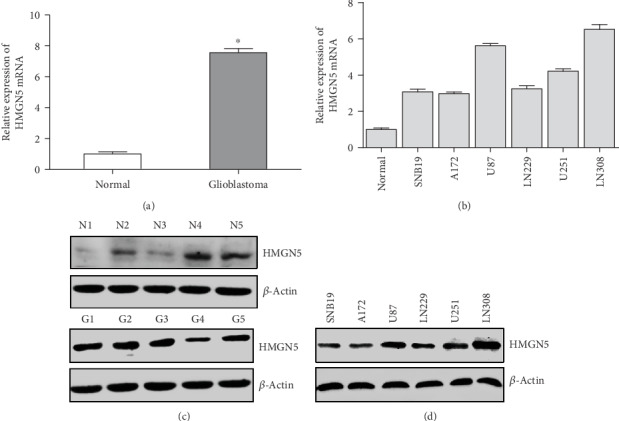
The expression of HMGN5 in human glioblastoma tissues and cell lines. (a) The relative expression of HMGN5 mRNA in 5 nontumoral brain tissues and 5 glioblastoma tissues was analyzed by RT-PCR. (b) The relative expression of HMGN5 mRNA in glioblastoma cell lines (U87, A172, SNB19, U251, LN229, and LN308) was analyzed by RT-PCR. (c) The relative expression of HMGN5 protein in nontumoral brain tissues and glioblastoma tissues was analyzed by western blot. (d) The relative expression of HMGN5 protein in glioblastoma cell lines by western blot.

RT-PCR and western blot were performed to detect the mRNA and protein levels of HMGN5 in tissues and glioblastoma cell lines (SNB19, A172, U87, LN229, U251, and LN308). As shown in Figures 1(a) and 1(c), both the mRNA and protein levels of HMGN5 in glioblastoma samples were higher than nontumoral brain tissues, and also the expression quantity of HMGN5 in the six glioblastoma cell lines was upregulated compared with that in nontumoral brain tissues (Figures 1(b) and 1(d)).

### 3.2. HMGN5 Knockdown Suppresses Glioblastoma Cell Proliferation and Induces Apoptosis

**Figure 2:**
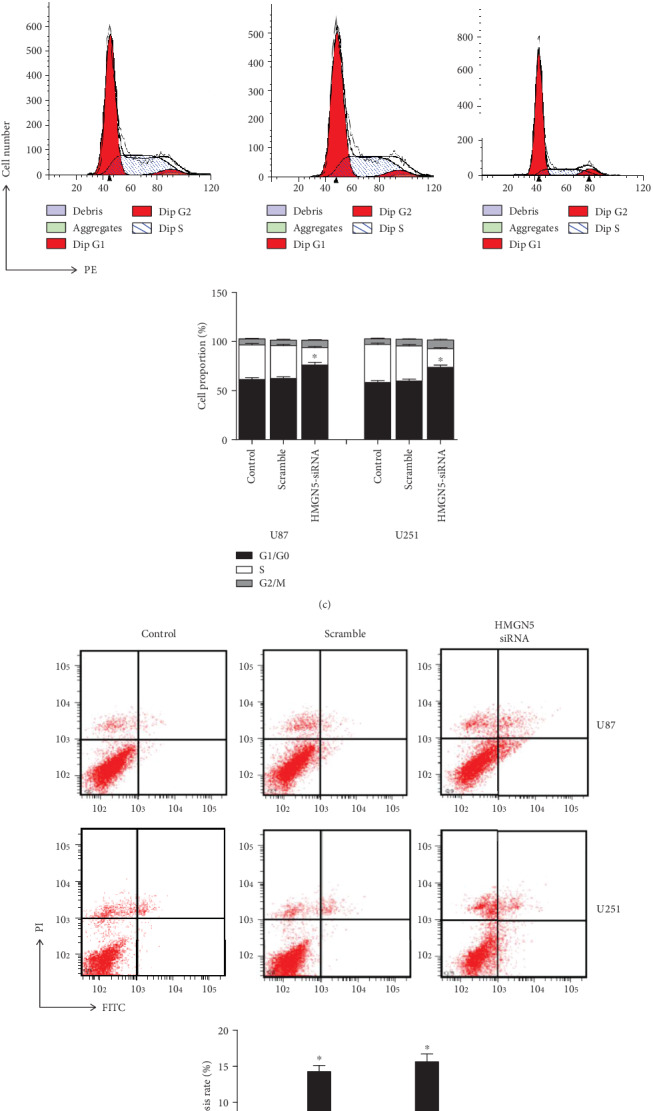
HMGN5 regulates the proliferation capacity, cell cycle progression, and cell apoptosis of glioblastoma cells. (a) The application of HMGN5 siRNA significantly reduced the levels of HMGN5 protein and mRNA levels in U87 and U251 cells (^∗∗^*p* < 0.01; ^∗^*p* < 0.05). (b) CCK8 assay detected that HMGN5 knockdown reduced the proliferation rate in U87 and U251 cells. (c) Flow cytometry assays were performed to analyze the cell cycle progression when the U87 and U251 cells were transfected with HMGN5 siRNA or scramble sequences (^∗∗^*p* < 0.01; ^∗^*p* < 0.05). (d) The result of flow cytometry analysis detected that apoptosis was increased after HMGN5 was knocked down in U87 and U251 cells (^∗∗^*p* < 0.01; ^∗^*p* < 0.05). (e) JC-1 staining was used to determine Δ*ψ*. JC-1 is normally visualized as green when Δ*ψ* is reduced (^∗∗^*p* < 0.01; ^∗^*p* < 0.05).

HMGN5-siRNA significantly downregulated the expression level of HMGN5 mRNA, and protein was decreased in U87 and U251 cells, respectively (Figure 2(a)).

HMGN5-siRNA inhibited the proliferation and increased the apoptosis rate of glioblastoma cells. CCK8 assays were used to investigate the effect of the interference of HMGN5 on glioblastoma cell proliferation, and the results indicated that the proliferation capacity was suppressed while being transfected with HMGN5-siRNA 48 hours (Figure 2(b)). FACS was used to detect the cell cycle and apoptosis. As shown in Figure 2(c), there was significant G0/G1 phase retardation in the HMGN5-siRNA group compared to the control group. Then, we stained the cells with the Annexin V-FITC apoptosis detection kit while being transfected with HMGN5-siRNA 48 hours. The results showed that the depression of HMGN5 induced the apoptosis of cells (Figure 2(d)). Moreover, the results of FACS also demonstrated that HMGN5 silence could trigger apoptosis, which included mitochondrial pathway apoptosis (Figure 2(e)); the mitochondrial membrane potential (Δ*ψm*) was lower in the HMGN5-siRNA group than in the two control groups. The result of the JC-1 assay indicated that HMGN5 was involved in early apoptosis in glioblastoma cell lines.

### 3.3. HMGN5 Knockdown Suppresses Cell Migration and Invasion

**Figure 3:**
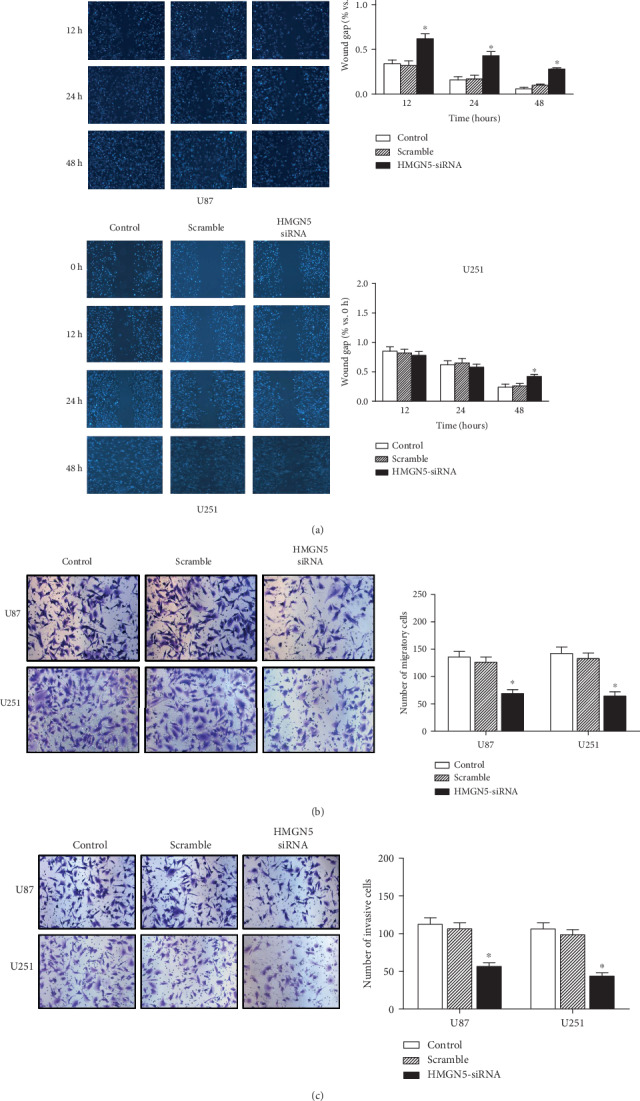
HMGN5 knockdown inhibits the migratory and invasive abilities of U87 and U251 cells. (a) Representative microscopy images of the scratch-wound-healing assay captured at 0 h, 12 h, 24 h, and 48 h. The mean level of migration distance observed in three random fields for each condition is shown in histograms. The wound-healing assay shows the cell migration capacity of glioblastoma cells after HMGN5 knockdown. (b and c) The Transwell assay was preformed to detect the invasion and migration of glioblastoma cells after HMGN5 knockdown (^∗∗^*p* < 0.01; ^∗^*p* < 0.05).

HMGN5-siRNA also decreased cell invasion and migration. Transwell assays and wound-healing assays showed that the HMGN5 knockdown group could significantly decrease the ability of migration (Figures 3(a) and 3(b)). And according to the Transwell assays, the HMGN5 knockdown group displayed a weaker capacity of invasion through an extracellular matrix than the scramble and control groups (Figure 3(c)).

### 3.4. HMGN5-siRNA Inhibits Glioblastoma Growth In Vivo

**Figure 4:**
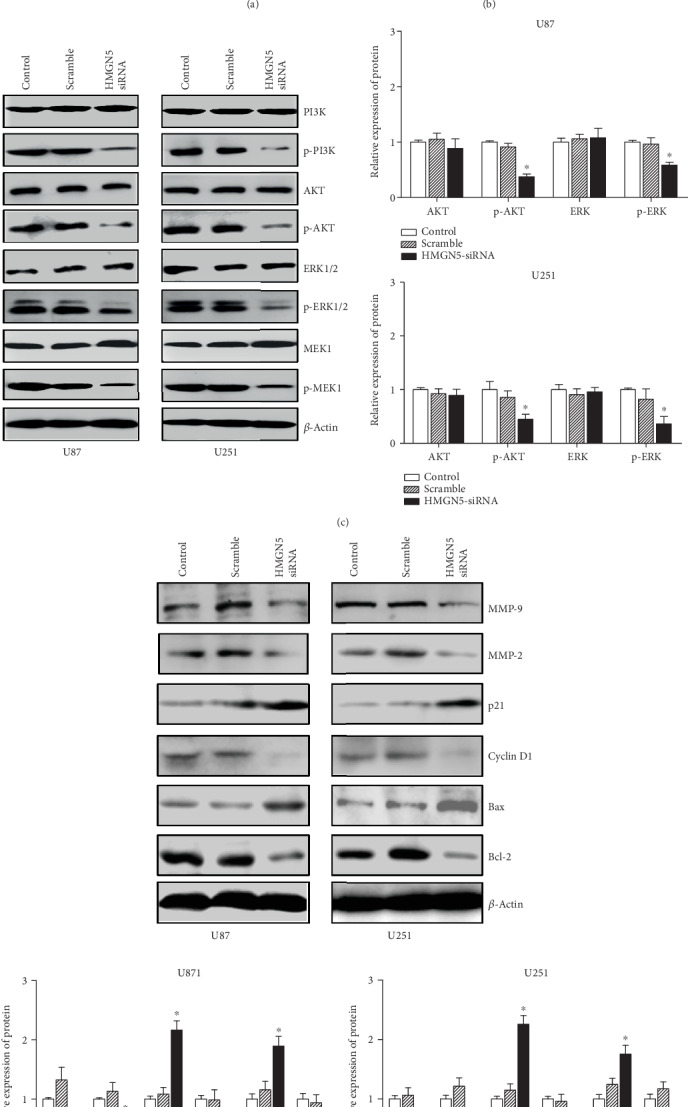
HMGN5 knockdown promotes xenograft U87 growth in vivo and the molecular mechanism related to malignant behaviors of glioblastoma cells in vitro. (a) The growth of the U87 xenograft was performed to confirm the effects of HMGN5 on tumor growth in vivo. (b) The photograph of the U87 xenograft tumor is shown at the end of a 21-day observation period. (c) The western blot results of Bcl-2, Bax, Cyclin D1, p21, MMP-2, and MMP-9 in indicated cells (^∗^*p* < 0.05). (d) The protein levels of t-ERK, p-ERK, t-AKT, and p-AKT in indicated cells (^∗^*p* < 0.05).

To further study the influence of HMGN5 knockdown in vivo, tumor xenograft models were established. As shown in Figure 4(a), the tumor growth rate diminished gradually when treated with HMGN5-siRNA at the 9th day compared to being treated with nonspecific sequences. And the mean tumor volume showed the largest difference from the nude mice models when being treated for 21 days (Figure 4(b)). These results indicated that HMGN5 knockdown suppressed the growth of glioblastoma.

### 3.5. HMGN5 Knockdown Is Related to the AKT and ERK Signaling Pathway

To further study the molecular mechanism of HMGN5 influencing glioblastoma, several proteins which are related to the malignant behavior of glioblastoma were detected by western blot. Knocking down HMGN5 decreased the expression of Bcl-2, Cyclin D1, MMP-2, and MMP-9 and increased the expression of Bax and p21 which is related to the malignant phenotype of tumor proliferation, migration, and invasion (Figure 4(c)).

Proteins which were involved in the AKT and MAPK signaling pathways were also measured by western blot. As shown in Figure 4(d), although there was no significant difference in AKT and ERK1/2 levels between the HMGN5-siRNA group and the control group, the phosphorylation of these four proteins in the HMGN5-siRNA group notably decreased.

## 4. Discussion

In this study, we confirmed that high-grade glioblastoma usually had higher expression of HMGN5 than lower grade glioblastoma, which suggested that a high level of HMGN5 is correlated to the malignancy of glioblastoma. To further investigate the function of HMGN5 in glioblastoma, we knocked down HMGN5 in two glioblastoma cell lines U87 and U251. Our findings demonstrated that HMGN5 was involved in regulating proliferation, apoptosis, migration, and invasion of glioblastoma cells. To investigate whether HMGN5 regulates the malignant behavior of glioblastoma cells via AKT and MEK oncogenic cascades, we examined AKT and ERK1/2 proteins.

Firstly, we demonstrated that the proliferative capacity of glioblastoma cells was decreased as HMGN5 was knocked down in vitro and in vivo. Then, FACS was performed to assess the role of HMGN5 in the cell cycle and cell apoptosis of glioblastoma. All the data above were consistent with previous research that HMGN5 promoted proliferation and inhibited apoptosis of glioblastoma [11]. Silencing HMGN5 could induce G2/M phase arrest with the downregulation of Cyclin B1 in bladder cancer cells [12] and prostate cancer cells [13]. While in our research, we found that HMGN5-siRNA arrested the cell cycle at the G1/G0 phase along with the decreased expression of Cyclin D1 and p21 protein in glioblastoma cells. Since HMGN5 was found to regulate mitochondrial pathway apoptosis and Bcl-2 family protein in prostate cancer cells [14], we also performed the JC-1 assay to evaluate the role of HMGN5 in the apoptosis of glioblastoma cells. As expected, HMGN5-siRNA induced mitochondrial pathway apoptosis in glioblastoma cells. Then, we examined the expression of Bcl-2 and Bax, which were key molecules involved in the regulation of the mitochondrial pathway of apoptosis [15]. Consistently, silencing HMGN5 decreased the expression of Bcl-2 and increased Bax. These results further confirmed that HMGN5 could regulate the mitochondrial apoptosis pathway of glioblastoma cells.

Studies showed that HMGN5 might promote migration and invasion of several types of cancer via regulating MMPs. MMP-2 or (and) MMP-9 was downregulated in breast cancer, osteosarcoma, and renal carcinoma cells with the silence of HMGN5 [16–18]. We performed Transwell and wound-healing assays to investigate the role of this gene in glioblastoma cells. The results demonstrated that silencing HMGN5 decreased the migratory and invasive capacity of U87 and U251 cells. Moreover, MMP-2 and MMP-9, the members of the metalloproteases family, which were frequently involved in glioblastoma invasiveness [19], were downregulated along with the reduction of HMGN5 in glioblastoma cells.

AKT and MEK signaling pathways are canonical signaling pathways whose aberrant activation is implicated in the malignant behavior of glioblastoma cells [20, 21]. Studies showed that the PI3K/AKT pathway was targeted by HMGN5 in human urothelial bladder cancer [9]. And our findings indicated that silencing HMGN5 downregulated p-PI3K and p-AKT in glioblastoma cells. As there were a series of downstream molecules of this pathway involved in proliferation, apoptosis, migration, and invasion of glioblastoma [19, 22], we examined some of the downstream molecules, including: Cyclin D1, p21, Bcl-2, Bax, MMP-2, and MMP-9. Previous studies showed that p-AKT could contribute to the G0/G1 phase retardation by upregulating the expression of Cyclin D1 and inhibiting its degradation [23]. And several molecules might regulate cell proliferation by targeting Cyclin D1 and p21 partially through PI3K/AKT [24]. Besides, the activated PI3K-dependent AKT could phosphorylate the Ser136/Ser112 residue of Bad, a member of the Bcl-2 family, which resulted in depolymerizing Bad with Bcl-2 or Bcl-XL. Then, the released Bcl-2 would exert its antiapoptotic effect. Similarly, Bax, one of proapoptotic proteins of Bcl-2 family, could be phosphorylated at its Ser184 residue to form heterodimers with Mcl-1 and Bcl-XL which could suppress cell apoptosis [25]. Taken together, we speculated that HMGN5 in glioblastoma cells could regulate Bcl-2, Bax, Cyclin D1, p21, MMP-2, and MMP-9 to play an oncogenic role partly via the PI3K/AKT pathway. Apart from the PI3K/AKT pathway, it was reported that HMGN5 could activate the MAPK signaling pathway to exert its function in prostate cancer cells [26]. Expectably, our research showed that knockdown of HMGN5 downregulated the expression of p-MEK1 and p-ERK1/2 in glioblastoma cells. Further studies are needed to make clear the mechanism how HMGN5 regulates the PI3K/AKT and MAPK pathways.

## 5. Conclusions

To summarize, our data validate that HMGN5 has a high expression in glioblastoma and functions as an oncogene. Also, HMGN5 knockdown regulates glioblastoma cell growth, invasion, and migration via the AKT and MAPK pathway. Our findings suggest that HMGN5 plays an important role in the initiation and progression of glioblastoma, which may provide us with an efficient target for glioblastoma-targeted therapy.

## Data Availability

The data used to support the findings of this study are available from the corresponding author upon request.
